# Enhanced Bacterial Wilt Resistance in Potato Through Expression of *Arabidopsis* EFR and Introgression of Quantitative Resistance from *Solanum commersonii*

**DOI:** 10.3389/fpls.2017.01642

**Published:** 2017-09-25

**Authors:** Federico Boschi, Claudia Schvartzman, Sara Murchio, Virginia Ferreira, Maria I. Siri, Guillermo A. Galván, Matthew Smoker, Lena Stransfeld, Cyril Zipfel, Francisco L. Vilaró, Marco Dalla-Rizza

**Affiliations:** ^1^Instituto Nacional de Semillas Canelones, Uruguay; ^2^Unidad de Biotecnología, Instituto Nacional de Investigación Agropecuaria Canelones, Uruguay; ^3^Departamento de Biociencias, Facultad de Química, Universidad de la República Montevideo, Uruguay; ^4^Departamento de Producción Vegetal, Centro Regional Sur, Facultad de Agronomía, Universidad de la República Canelones, Uruguay; ^5^The Sainsbury Laboratory, Norwich Research Park Norwich, United Kingdom; ^6^Programa de Producción Hortícola, Instituto Nacional de Investigación Agropecuaria Canelones, Uruguay

**Keywords:** *Ralstonia solanacearum*, bacterial wilt, *Solanum tuberosum*, *Solanum commersonii*, pattern recognition receptor, EFR, quantitative resistance

## Abstract

Bacterial wilt (BW) caused by *Ralstonia solanacearum* is responsible for substantial losses in cultivated potato (*Solanum tuberosum*) crops worldwide. Resistance genes have been identified in wild species; however, introduction of these through classical breeding has achieved only partial resistance, which has been linked to poor agronomic performance. The *Arabidopsis thaliana* (At) pattern recognition receptor elongation factor-Tu (EF-Tu) receptor (EFR) recognizes the bacterial pathogen-associated molecular pattern EF-Tu (and its derived peptide elf18) to confer anti-bacterial immunity. Previous work has shown that transfer of AtEFR into tomato confers increased resistance to *R. solanacearum*. Here, we evaluated whether the transgenic expression of *AtEFR* would similarly increase BW resistance in a commercial potato line (INIA Iporá), as well as in a breeding potato line (09509.6) in which quantitative resistance has been introgressed from the wild potato relative *Solanum commersonii.* Resistance to *R. solanacearum* was evaluated by damaged root inoculation under controlled conditions. Both INIA Iporá and 09509.6 potato lines expressing *AtEFR* showed greater resistance to *R. solanacearum*, with no detectable bacteria in tubers evaluated by multiplex-PCR and plate counting. Notably, AtEFR expression and the introgression of quantitative resistance from *S. commersonii* had a significant additive effect in 09509.6-AtEFR lines. These results show that the combination of heterologous expression of *AtEFR* with quantitative resistance introgressed from wild relatives is a promising strategy to develop BW resistance in potato.

## Introduction

Bacterial wilt (BW) caused by *Ralstonia solanacearum* is considered as one of the most destructive bacterial diseases of plants (Mansfield et al., 2012). The disease has a worldwide distribution, affecting crop production in tropical, subtropical, and temperate regions, where cold-tolerant strains were introduced (Martin and French, 1985; Muthoni et al., 2012). *R. solanacearum* is able to persist in soil, weed, plant debris, rhizospheres, and alternate hosts. It can be spreaded trough irrigation water or infected planting material hindering pathogen eradication (Hayward, 1991; Laferriere et al., 1999; Liu et al., 2016). When living freely in the soil, *R. solanacearum* occurs in a motile form to efficiently find and invade the host which locates through chemotaxis and aerotaxis (Yao and Allen, 2006, 2007). *R. solanacearum* generally enters through wounded roots or natural openings and it infects the intercellular space of the root cortex and vascular parenchyma. It can also invade xylem vessels and disseminate to the stem and leaves, where bacterial cell density can reach up to 10^9^ cfu⋅g^-1^ of host tissue (Yao and Allen, 2006; Álvarez et al., 2010). At this stage, a cell density-dependent (i.e., quorum sensing) conversion occurs from a motile phenotype to a non-motile virulent phenotype, adapted to a plant parasitic lifestyle (Tans-Kesrten et al., 2004).

More than 200 plant species are affected by *R. solanacearum*, including some economically important crops such as potato, tomato, tobacco, and banana (Hayward, 1991). Chemical control of BW is ineffective, the use of healthy seeds and pathogen-free soil and water, as well as crop rotation, are the principal means of control (Álvarez et al., 2010). In regions where the pathogen is endemic, the use of partially resistant varieties is one of the main strategies to control the pathogen. Although loci providing quantitative resistance have been identified in tomato (Mangin et al., 1999; Wang et al., 2000; Carmeille et al., 2006), tobacco (Qian et al., 2013), and eggplant (Lebeau et al., 2013), there are not resistant commercial varieties against *R. solanacearum* available (Huet, 2014).

Potato (*Solanum tuberosum*) is widely cultivated across the world, and is regarded as the fourth most important food crop and one of the most significant solanaceous crops (Liu et al., 2016). For potato grown in the tropics, BW is one of the most important biotic constraints to production after late blight caused by the oomycete *Phytophthora infestans* (Priou et al., 1999). The use of resistant crops is a cost-effective and environmentally friendly form of control, but few sources of resistance are available in the potato germplasm (Laferriere et al., 1999). Wild potato species related to *S. tuberosum* have been used in order to generate resistant potato cultivars (Sequeira and Rowe, 1969; Carputo et al., 1997; Narancio et al., 2013). BW resistance has, for example, been reported in *Solanum stenotomum* (Fock et al., 2001), *S. chacoense* (Chen et al., 2013), *S. phureja* (Sequeira and Rowe, 1969; French and De Lindo, 1982; Fock et al., 2000), and *S. commersonii* Dun (Laferriere et al., 1999; Kim-Lee et al., 2005; Carputo et al., 2009; González et al., 2013). Particularly, *S. commersonii* is a wild diploid, tuber-bearing species native to Uruguay, East Argentina, and South Brazil. It shows high genetic diversity (Pianzzola et al., 2005; Siri et al., 2009) with desirable traits like tolerance to low temperatures and resistance to various pathogens, including *R. solanacearum.* Although hybridization of *S. commersonii* × *S. tuberosum* is hampered by complex interspecific crossing barriers, hybrids with partial resistance to BW were obtained using different strategies for ploidy manipulation (Kim-Lee et al., 2005; Carputo et al., 2009; Guidot et al., 2009; González et al., 2013; Narancio et al., 2013; Zuluaga Cruz et al., 2014).

The immune system of plants senses and responds effectively against most potential pathogens. Plasma membrane-localized pattern recognition receptors (PRRs) recognize pathogen-associated molecular patterns (PAMPs), as well as endogenous elicitors released during infection. PRR activation triggers a rapid intracellular signaling cascade leading to both local and systemic immune responses (Boller and Felix, 2009). In most cases, PAMP-triggered immunity (PTI) is capable to avoid disease development contributing to plants non-host resistance (Lee et al., 2016). The elongation factor-Tu (EF-Tu) receptor (EFR) from *Arabidopsis thaliana* (AtEFR) is a PRR that recognizes the bacterial PAMP EF-Tu (or the conserved *N*-acetylated epitope elf18, composed by the first 18 amino acids of EF-Tu) (Kunze, 2004; Zipfel et al., 2006). In fact, elf18 recognition is restricted to the plant family *Brassicaceae* (Kunze, 2004; Zipfel et al., 2006; Boller and Felix, 2009). Transgenic expression of *AtEFR* in other plant families has shown to confer elf18 perception and to increase resistance to adapted bacterial pathogens, which suggested that interfamily transfer of plant PRRs can be used to engineer disease resistance in crops (Lacombe et al., 2010; Lu et al., 2015; Schoonbeek et al., 2015; Schwessinger et al., 2015b; Zipfel and Oldroyd, 2017). In particular, expression of *AtEFR* in *Nicotiana benthamiana* and tomato (*S. lycopersicum* variety Moneymaker), both members of the *Solanaceae* family, conferred resistance to non-related phylogenetically bacterial pathogens such as *Pseudomonas syringae* pv. *tabaci* or *R. solanacearum* (Lacombe et al., 2010). Moreover, expression of AtEFR conferred elf18 recognition in monocotyledonous crop species such as rice and wheat (Schoonbeek et al., 2015; Schwessinger et al., 2015a).

In this work, we evaluated the effect of *AtEFR* gene expression in a commercial potato line (INIA Iporá) and in an interspecific breeding line (09509.6), into which quantitative resistance to BW from the wild relative *S. commersonii* has been introgressed. We report enhanced resistance to *R. solanacearum* in both potato genotypes expressing *AtEFR*, interestingly, greater survival rates and reduced disease symptoms were observed in 09509.6-AtEFR compared with INIA Iporá-EFR lines. For both genetic backgrounds the expression of the *AtEFR* gene controlled bacterial cell population, thus preventing the conversion to the pathogenic phenotype. These results show that the combined heterologous expression of *AtEFR* with quantitative resistance introgressed from wild relatives is a promising strategy to develop BW resistance and contribute to an integrated disease control in potato.

## Materials and Methods

### Bacterial Strains and Growth Conditions

*Ralstonia solanacearum* strain UY031 (race 3, biovar 2A/phylotype IIB, sequevar 1) (Siri et al., 2011) was grown at 28°C in Kelman medium supplemented with 2,3,5-trifenil tetrazolium chloride (TTC). To prepare inocula, bacteria were grown overnight in nutrient broth at 28°C with shaking at 200 × *g*. Cells were pelleted by centrifugation, suspended in water, and spectrophotometrically adjusted to 10^7^ cfu⋅mL^-1^.

### Plant Material and Growth Conditions

*Solanum tuberosum* cv INIA Iporá (susceptible to BW) and the partially resistant clone 09509.6 were used for plant transformation. Partial resistance against *R. solanacearum* was introgressed from a *S. commersonii* accession and was achieved through sexual polyploidization (2*n* gametes) as part of the INIA potato breeding program, proving to be heritable through several breeding generations (Gaiero et al., 2017). The crossing scheme involved *S. phureja* as a bridge species followed by two successive backcrosses to *S. tuberosum*, at the National Institute for Agricultural Research (INIA) (Dalla-Rizza et al., 2016). The BC2 clone 09509.6 was selected as a promising resistant candidate (González et al., 2013). Plants were propagated as previously described (Cruz et al., 2014). Briefly, *in vitro* single-node pieces growing on Murashige and Skoog (MS) agar medium were supplemented with sucrose at 30 g⋅L^-1^, and maintained at 22°C with a cycle of 16 h of light and 8 h of darkness. After 2 weeks, plantlets were transferred into pots containing TREF soil mix (Tref Substrates BV, Moerdijk, Netherlands) and grown for 1 week in a greenhouse at 22–25°C within plastic boxes with more than 90% RH. Then, they were grown for an additional week in a growth chamber at 27°C and 65% RH with a photoperiod of 12 h. For long-term (up to 9 months) *in vitro* maintenance of the INIA Iporá-EFR and clone 09509.6-EFR, plants were kept at 22°C with a cycle of 16 h of light and 8 h of darkness in a preservation medium (20 mL of MS without vitamins, sucrose 25 g⋅L^-1^, D-sorbitol 40 g⋅L^-1^, agar 8 g⋅L^-1^, pH 5.8).

### Generation of AtEFR Transgenic Potato Lines

Potato internode segments were used as the target tissue for transformation with *Agrobacterium tumefaciens* using the 35S::EFR-HA construct cloned into pBIN19. Briefly, internode sections were harvested from 6-weeks-old axenically grown plants and propagated in MS medium. These internode sections were inoculated with an *A. tumefaciens* suspension (strain AGL1 harboring the appropriate binary vector), made by diluting 100 μL of an overnight bacterial culture in to 20 mL of MS broth, 3% sucrose, pH 5.7 for 20 min, shaking in the dark at 60 × *g*. Inoculated explants were then blotted dry and placed on to MS medium, containing 2.0 mg⋅L^-1^ zeatin riboside, 0.2 mg⋅L^-1^ naphthalene-acetic acid (NAA), 0.02 mg⋅L^-1^ gibberellic acid (GA_3_) in a growth room (24°C constant, 16 h light), shaded under a piece of paper. After 3 days, the internode sections were transferred to the same medium for co-cultivation with 320 mg⋅L^-1^ ticarcillin disodium/potassium clavulanate and 100 mg⋅L^-1^ kanamycin and placed in to the light. Explants were transferred to fresh media every fortnight until resistant callus formed at the cut ends of the internode sections (about 6 weeks). Explants were then transferred to the same medium, with a concentration of NAA 10 times lower. As shoots formed (2–4 weeks), they were excised and placed in to rooting medium (MS medium, 2% sucrose, 100 mg⋅L^-1^ myoinositol, 2.0 mg⋅L^-1^ glycine, pH 5.7, 0.2% Gelrite with 320 mg⋅L^-1^ ticarcillin disodium/potassium clavulanate, and 100 mg⋅L^-1^ kanamycin). Transformed plants quickly developed roots in the presence of kanamycin and were selected for further screening.

The presence of the *AtEFR* gene in transformed potato lines of both genotypes was confirmed by PCR. Genomic DNA was isolated using the ZR Plant/Seed DNA MiniPrep Kit (Zymo Research) following manufacturer’s instructions. The PCR mix for amplification contained Taq Buffer 1× (Thermo), MgCl_2_ 2.5 μM, dTNPs 0.5 μM, primers 0.4 μM, and Taq polymerase 1U (Invitrogen) in a final volume of 20 μL. The cycling conditions were: 94°C 3 min (initial denaturation), 94°C for 30 s (denaturation), 60°C for 40 s (annealing), 72°C for 40 s (extension) for 35 cycles, with a final extension at 72°C for 2 min. *AtEFR* internal primers were used (**Table 1**) and PCR products were analyzed by electrophoresis in 1–2% agarose gels.

**Table 1:** Primers and probes used

Primer/probe	Sequence (5′–3′)
**Genotyping**	
AtEFR1-Fw	CCA GTT TAG TTC TGC TGG TGT CA
AtEFR1-Rv	GTT GGCCTC CCA TTC CAT ACT
**Gene copy number**	
AtEFR-Fw	CCA GTT TAG TTC TGC TGG TGT CA
AtEFR-Rv	GTT GGC CTC CCA TTC CAT ACT
AtEFR-Pr	6-FAM- CCA TTG GCT ATG CCG CGC CA- TAMRA
UDP-Fw	GGA CAT GTG AAG AGA CGG AGC
UDP-Rv	CCT ACC TCT ACC CCT CCG C
UDP-Pr	6-FAM-CTA CCA CCA TTA CCT CGC ACC TCC TCA-TAMRA

Phenotypic characterization of potato plants was done according to UPOV Guidelines for the conduct of tests for distinctness, uniformity, and stability of potato varieties (International Union For The Protection Of New Varieties Of Plants [UPOV], 2004) on 90-day potato plants grown in greenhouse conditions to tuberization (20 ± 2°C and 10 h light).

### Copy Number Determination

Copy number of the *AtEFR* gene in transformed potato lines was determined by qPCR. DNA was purified from 3- to 4-week-old leaves using ZR Plant DNA MiniPrep (Zymo Research) following manufacturer’s instructions. UDP-glucose pyrophosphorylase (UGPase) was used as a single-copy reference gene (Delobel et al., 2008). Samples were serially diluted starting at 35 ng⋅μL^-1^ and amplified by triplicate using a TaqMan^®^ Master Mix (Applied Biosystem), 200 nM of each primer (**Table 1**), 100 nM label probes, and 100 ng DNA to a final volume of 25 uL on an ABI 7500 Applied Biosystem Real-Time System (United States). Reaction efficiency was calculated for each standard curve according to *E* = 10ˆ(-1/pend)-1. *AtEFR* gene copy number was calculated as the ratio between the cp of AtEFR and UGPase genes.

### Western Blot

Total protein extracts were obtained from 3- to 4-week-old leaves ground to fine powder in liquid nitrogen and boiled at 95°C for 5 min in Sample buffer [65.8 mM Tris–HCl Buffer, pH 6.8, 25% (w/v) glycerol, 2% SDS, 0.01% (w/v) bromophenol blue, and 5% (w/v) 2-mercaptoethanol]. Proteins were separated on 8% SDS-PAGE gels and electroblotted onto PVDF membranes (Biorad Mini Trans-Blot^®^ Electrophoretic Transfer Cell). Membranes were blocked in 5% (w/v) BSA in PBS overnight at 4°C. Anti-HA antibody (Roche) was diluted in 0.5% (w/v) BSA in PBS–Tween 0.1% (w/v) solution to 1:1000 and incubated for 1 h at room temperature. Secondary anti-rat-HRP (Sigma) was diluted 1:2000 and incubated 1 h at room temperature as well. Bands were visualized using chemiluminescent substrate SuperSignal West Femto Kit (Thermo) before exposure to film (Biomax light film Sigma–Aldrich). Membranes were stained with Coomassie Blue to check for equal loading (Welinder and Ekblad, 2011). Samples from four plants were pooled, experiments were performed twice.

### ROS Burst Assay

Disks (4 mm diameter) from up to 4-week-old leaves from INIA Iporá AtEFR or clone 09509.6 AtEFR plants were sampled and placed in 96-well white microwell plates (Thermo Scientific) containing 200 μL of distilled water for 16 h at room temperature. The following day, water was replaced with 17 μg⋅mL^-1^ (w/v) luminol (Sigma), 10 μg⋅mL^-1^ horseradish peroxidase (Sigma), and 100 nM elf18 peptide (GenScript) solution. Luminescence was immediately measured over 60 min using a Varioskan Flash Multiplate Reader (Thermo Scientific). A solution without elf18 peptide was used as a negative control. Experiment sample size was *n* = 16 and repeated twice with similar results.

### Plant Inoculation and Disease Rating

Response assays to *R. solanacearum* were performed with 4-week-old acclimatized plants in 88-well seedbeds with 4 g of pathogen-free substrate per plant. Plants were drench-inoculated with a 1 × 10^7^ cfu⋅mL^-1^ bacterial suspension to a final concentration of 2.5 × 10^6^ cfu⋅g^-1^ of substrate. Prior to inoculation, roots were lightly wounded to facilitate infection by making a 2 cm deep hole in the soil of each pot with a pipette tip. Plants were placed in a controlled conditions chamber at 28 ± 2°C with a cycle of 14:10 h of light:darkness. Four replicates of 16 plants of each genotype were inoculated in a randomized complete block design in two independent experiments. Non-transformed plants were inoculated for disease rating comparison. Plants of each genotype mock-inoculated with water were used as negative controls. Disease development was evaluated weekly using a disease index scale ranging from 0 (no wilting symptoms) to 4 (all leaves wilted-dead plant) up to the fourth week after inoculation (Winstead and Kelman, 1952; Cruz et al., 2014). Pathogenicity was estimated by the area under the disease progression curve (AUDPC) based on the average wilt score over time. The AUDPC was calculated as the integral of the disease index of each treatment in the graph that relates the disease level versus time. Analysis of variance was performed to evaluate for significance differences between treatments, between genotypes, and between the presence or absence of the *AtEFR* transgene. Tukey’s test was used for analysis between treatments. Statistical analyses were performed using Infostat software (Di Rienzo et al., 2011).

In tuberization assays aiming to evaluate the transmission of *R. solanacearum* to the new tubers, 11 plants per event were inoculated as previously described on 1 L pots. Plants were kept at 28 ± 2°C with a cycle of 14 h of light for 14 days. After that, in order to promote tuberization, conditions were changed to 20 ± 2°C and 10 h light during 90 days. Tubers from resistant plants were harvested and analyzed for *R. solanacearum* infection. Next, these tubers were used as seeds grown at the same conditions as before. After 90 days, BW development was evaluated on the tubers harvested from resistant lines.

### Detection of Latent *R. solanacearum* Infections

Detection of latent *R. solanacearum* infection in plants without symptoms was performed by BIO-multiplex PCR. Briefly, 2-cm stem samples from asymptomatic plants were washed with water, dried, treated with sodium hypochlorite 1% (v/v) for 1 min, washed with sterile water, and ground in extraction buffer. Tubers were water washed, disinfected, and 1 cm^3^ samples were cut near the stolon area and treated as described previously. Each sample was plated by triplicate in mSMSA selective media and incubated for 48 h at 28°C. Growing pin-point colonies from two of the plates were collected with sterile water and heat-lysed for 20 min at 99°C. The third plate was incubated for additional 5 days and used for colony count. BIO-multiplex PCR was performed using generic *R. solanacearum* primers 759/760 (Opina et al., 1997) and specific primers for phylotype IIB sequevar 1 strains (unpublished data). In tuber assays four plants of each genotype were analyzed, whereas in tuber identification, three tubers from each resistant plant were pooled and evaluated. Experiments were repeated twice.

## Results

### Generation of AtEFR Potato Transgenic Lines

The effect of the expression of *AtEFR* in potato on BW resistance was evaluated in two different genetic backgrounds: a commercial susceptible variety INIA Iporá and a breeding clone 09509.6 that has partial BW resistance. In clone 09509.6 genes from the wild potato species *S. commersonii* have been introgressed using the bridge species *S. phureja* and stabilized genetically through backcrossing. Primary transformed potato plants were selected in tissue culture by their ability to regenerate in the presence of kanamycin (see “Materials and Methods”). Since potato plants are propagated clonally, no homozygous selection was performed with the events. In total 38 primary independent transgenic potato lines were generated in INIA Ipora and 12 primary independent transgenic lines were generated in 09509.6. We selected the transgenic plants INIA Iporá AtEFR 3, 12, and 27, and clone 09509.6 AtEFR 34, 37, and 41 for further analysis and resistance assays.

**FIGURE 1:**
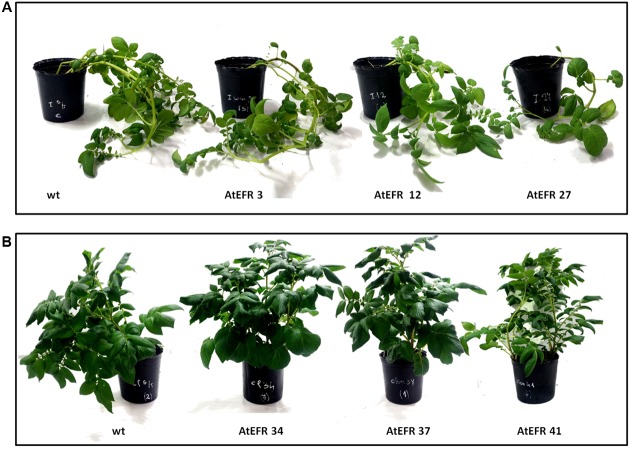
AtEFR potato plants are phenotypically similar to non-transformed plants. Representative 90-day-old plants grown from tubers in greenhouse. **(A)** INIA Iporá. **(B)** clone 09509.6. No differences were observed in controlled conditions between wild-type and transformed plants at phenotypic level.

Transformed *AtEFR* plants were phenotypically similar to wild-type plants. No difference was observed in stem pigmentation, size, openness, leaflets, pigmentation, and shape of leaves; coalescence, waviness of margin, depth of veins, and glossiness of leaflets in *AtEFR* plants compared to wild-type plants grown in controlled-environment chambers (**Figure 1**).

### Transgenic Expression of *AtEFR* in Potato Confers elf18 Responsiveness

**FIGURE 2:**
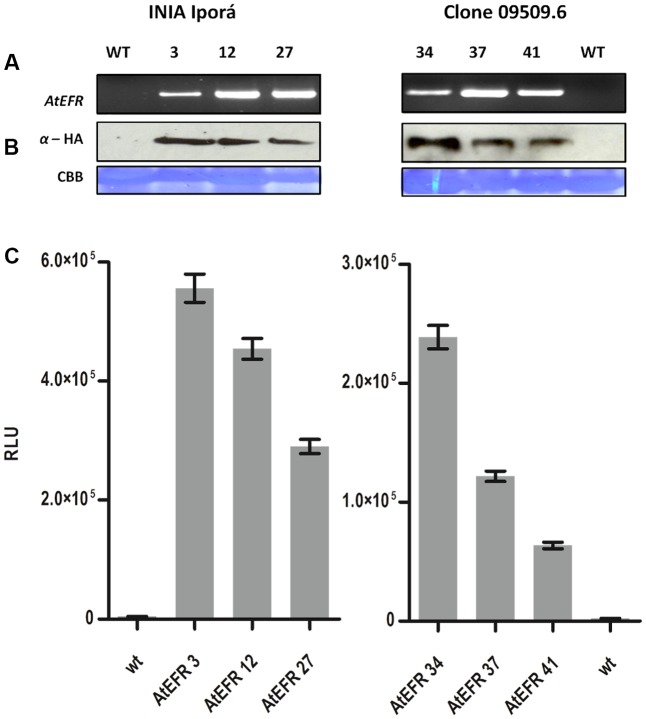
*At*EFR expression in potato confers elf18 responsiveness. **(A)** *AtEFR* gene detection by PCR of transgenic potato lines. **(B)** Protein expression of *At*EFR detection by Western blot. Upper panel anti-HA blot; lower panel CBB membrane staining as loading control. **(C)** ROS production triggered by 100 nM elf18 in INIA Iporá and clone 09509.6 leaf discs measured as RLU over a period of 60 min. Results are average ± standard error (*n* = 16). Experiments were performed twice with similar results.

Transformed potato plants were genotyped by PCR for *AtEFR* presence (**Figure 2A**), while gene copy number was determined by qPCR (**Table 2**). INIA Iporá AtEFR 3, 12 and clone 09509.6 AtEFR 27 had a single copy, whereas INIA Iporá AtEFR 27 had two copies while clone 09509.6 AtEFR 37 and 41 had three gene copies. These results are related to the transformation method used, as with *Agrobacterium* transformation, neither insertion site nor transgene copy number is controlled.

**Table 2:** *AtEFR* gene copy number of potato lines determined by qPCR.

INIA Iporá	Copy number	clone 09509.6	Copy number
3	1	34	1
12	1	37	3
27	2	41	3
wt	0	wt	0

Expression of AtEFR-HA was evaluated at the protein level by western blot on positively genotyped plants. All potato lines expressed full-length AtEFR to a similar level (**Figure 2B**). Even though the molecular weight observed for AtEFR protein is higher than the predicted, this observation was also made in *Arabidopsis* and is related to glycosylation (Häweker et al., 2010). Next, we evaluated whether AtEFR expressed in the different potato genotypes could recognize its ligand elf18 inducing a rapid production of reactive oxygen species (ROS). As shown in **Figure 2C**, whereas wild-type INIA Iporá and clone 09509.9 plants were insensitive to elf18, transgenic AtEFR plants produced ROS in response to elf18. Notably, the response in Iporá events was two times greater than in clone 09509.6 AtEFR. These results show that the two transformed genetic backgrounds are able to recognize elf18; thus, INIA Iporá AtEFR and clone 09509.6 AtEFR expressed a functional AtEFR receptor.

### Transgenic Expression of *AtEFR* in Potato Confers Enhanced Bacterial Wilt Resistance

**FIGURE 3:**
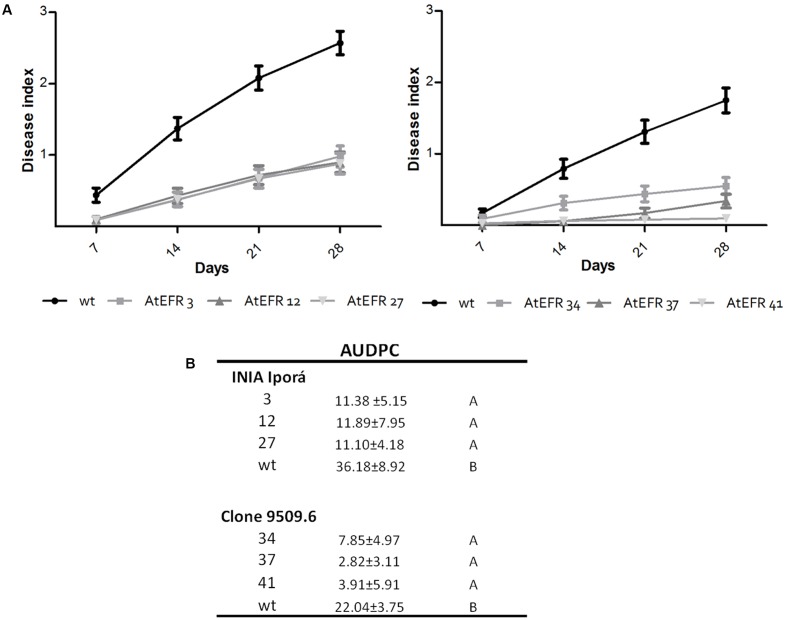
Transgenic expression of *AtEFR* in potato confers BW resistance. **(A)** BW progress curves on INIA Iporá (left) and clone 09509.6 (right) after soil inoculation with *R. solanacearum* strain UY031. The mean of two experiments are represented in each point. **(B)** AUDPC values for the average wilting score ± SD as means of two independent experiments. Data were pooled across trials of repeated experiments because no significant effects involving trials were found in the analyses of variance. Transformed AtEFR potato plants of both genotypes responded statistically different when compared to wild-type controls, as assayed by ANOVA (*p* < 0.001). Tukey’s HSD test as means with different letters are significantly different (*p* < 0.05).

Once characterized, the AtEFR potato lines were used to evaluate whether this PRR could provide BW resistance. To this end, soil-drench inoculation was performed and the development of wilting symptoms was measured every 7 days for 28 days (**Figure 3A**). Aggressiveness was assessed by calculating the AUDPC (**Figure 3B**). In INIA Iporá AtEFR plants, AUDPC values were at least three times lower than in wild-type plants (*p* < 0.0001). All events behaved as a single group as assessed by Tukey’s statistical analysis (**Figure 3B**). There was no evidence that copy number of *AtEFR* gene correlated with disease response, since genotypes with one or three copies responded equally. As expected, clone 09509.6 wild type showed greater resistance to *R. solanacearum* when compared with INIA Iporá wild type. Moreover, clone 09509.6 AtEFR events had significantly lower AUDPC values when compared with wild type (*p* < 0.0001), and, again, no correlation between copy number and response was observed. When mean AUDPC scores were compared between both genotypes, breeding clone genotypes had statistically lower disease ratings. In this sense, clone 09509.6 AtEFR 37 was the most resistant event evaluated. This analysis showed that *AtEFR* expression contributed to resistance to *R. solanacearum* in both potato lines in spite of the different genetic backgrounds. Moreover, in 09509.6 AtEFR lines the response observed was enhanced, probably due to the combined effect with introgressed quantitative genes from *S. commersonii.*

### Reduced Occurrence of Latent Infections in AtEFR Potato Lines

**FIGURE 4:**
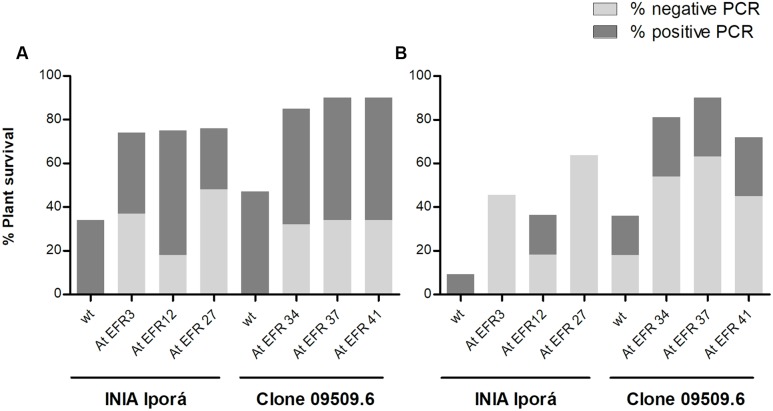
Occurrence of latent infections is reduced in AtEFR potato lines. **(A)** Latent infection in stems. Bars indicate percentage of plant survival after inoculation (28 d.p.i.). The proportion of positive replicates by PCR to *R. solanacearum* are denoted in dark gray, while negative for bacterial presence is denoted in light gray. Data samples correspond to averages of two independent experiments (*n* = 4). **(B)** Latent infection in tubers. Bars indicate percentage of plant survival (90 d.p.i.), while the proportions of positive replicates by PCR to *R. solanacearum* are denoted in dark gray and negative tubers in light gray.

Latent (i.e., symptomless) *R. solanacearum* infection is a major problem in BW control by loss of market value, quarantine measures, and is an impediment to the use of tuber as potato seeds. To determine latent infection of resistant plants from inoculation assays, we tested bacterial presence in stems by BIO-multiplex PCR and plate counting. The percentage of plant survival 28 days post-inoculation per genotype is indicated in **Figure 4A**, as well as the proportion of positive *R. solanacearum* plants. AtEFR INIA Iporá and clone 09509.6 AtEFR events showed a reduction in the percentage of infected stems compared to wild-type plants. Bacterial counts in all positive transformed replicates were similar in bacterial load; an average of 10^3^ cfu⋅mL^-1^ was detected in INIA Iporá AtEFR, while clone 09509.6 AtEFR events had an average of 10^2^ cfu⋅mL^-1^ (**Figure 4A** and Supplementary Material).

In cultivated areas, symptomless infected tubers used as potato seeds are an important mechanism of BW spread. In order to determine if *R. solanacearum* could persist in tubers from inoculated asymptomatic AtEFR plants, we performed an independent experiment in which inoculated potato plants were able to tuberize. Tubers from resistant, symptomless plants were harvested and bacterial presence was determined. As shown previously, AtEFR plants were more resistant to *R. solanacearum* than wild-type plants, where clone 09509.6 AtEFR showed higher survival percentages (**Figure 4B**). When tubers were analyzed, AtEFR plants had lower proportion of positive tubers when compared to wild-type plants (**Figure 4B**). Particularly, INIA Iporá AtEFR 3 and 27 had less than 10 cfu⋅mL^-1^ since no bacteria by multiplex PCR or plate counting was detected (**Figure 4B**). On the other hand, clone 09509.6 AtEFR had an average of 10^2^ cfu⋅mL^-1^ in stem tissues. Taken together, these results show that the response to AtEFR potato plants toward BW is enduring up to tuberization, where transformed plants were more resistant to *R. solanacearum* than wild-type plants. Even though latent infection was detected in stems and tubers, AtEFR plants had lower percentage of positive plants and bacterial loads determined were equal or less than 10^3^ cfu⋅mL^-1^.

Finally, we wanted to test if tubers infected with *R. solanacearum* from the previous assay would produce BW symptoms or disseminate the disease throw tubers from the next harvest. To this purpose, tubers tested in tuberization assay were used as seeds. Interestingly, none of the plants showed BW symptoms (data not shown). Moreover, in the tubers harvested and assayed by multiplex PCR and plate counting no bacteria was detected by PCR, indicating a concentration less than 10 cfu⋅mL^-1^. In this assay no differences were observed between wild-type and transformed plants.

## Discussion

Enhancing the genetic resistance of potato to *R. solanacearum* has been proposed to be the most economical, social, and effective approach for controlling BW (Jones et al., 2014). However, breeding for BW resistance has several challenges, as durable resistance in combination with desirable agronomic traits, and adaptation to different agro-ecological zones and the genetic variability of the pathogen are needed. Moreover, dissemination due to tolerant plants should be avoided, while availability of sources of resistance and transferring high number of genes may be difficult and can be linked to undesirable traits (Denny, 2006; Huet, 2014). *S. commersonii* has been explored as a source of quantitative resistance for BW. This wild potato species, endemic in Uruguay, shows high resistance to BW (Laferriere et al., 1999; Carputo et al., 2009).

Interfamily transfer of plant PRRs is showing to be an interesting strategy to engineering broad-spectrum disease resistance that may be durable (Zipfel and Oldroyd, 2017). In fact, heterologous expression of *AtEFR* in *N. benthamiana*, tomato, rice, and wheat has shown to enhance response to several pathogenic bacteria (Lacombe et al., 2010; Zipfel, 2014; Schoonbeek et al., 2015; Schwessinger et al., 2015a). Moreover, expressing *AtEFR* offers several advantages over the current alternatives to improving resistance to *R. solanacearum*. Using the plant’s own immune system to combat plant diseases constitutes a legitimate plant response preferable to the use of agrochemical inputs, which pose health risks and are economically and environmentally unsustainable (Lacombe et al., 2010). This approach to creating resistant varieties may also reduce the spread of the pathogen through infected/infested seed tubers.

We chose two contrasting genotypes (susceptible versus partially resistant to BW) for genetic transformation in order to compare the effect of *AtEFR* expression. In greenhouse conditions, transformed plants were phenotypically similar to wild types, indicating that the transformation process and the insertion site had no deleterious effects. Nevertheless, characterization of plant performance under field conditions will be necessary to determine other agronomic important characteristics such as plant cycle and tuber production. In the case of the commercial variety INIA Iporá, field trials will be needed to determine if agronomic characteristics other than AtEFR-mediated effect are maintained.

It is interesting to note that INIA Iporá transformation produced more single-copy events (70%) than clone 09509.6, in which up to 12 copies of the gene were observed (data not shown). Differences in plant genes involved in *Agrobacterium*-mediated transformation may explain the different outcome for a cultivated potato and an interspecific clone (Gelvin, 2003). We selected events with one to three *AtEFR* copies for further analysis. We confirmed AtEFR protein expression as well as elf18 responsiveness by measuring ROS production. Thus, transgenic potato lines expressing *AtEFR* under the control of the constitutive 35S promoter expressed a functional AtEFR receptor. These results indirectly showed – as previously demonstrated for other *Solanaceae* species (Lacombe et al., 2010) – that immune signaling components downstream of AtEFR are conserved in potato. This is interesting considering that the divergence of solanaceous species from the progenitor from *A. thaliana* dates back 112–156 million years ago, whereas *S. tuberosum* and *S. commersonii* diverged only 3 million years (Carvallo et al., 2011).

Next, transgenic potato plants expressing *AtEFR* were tested to evaluate whether EF-Tu responsiveness was associated with increased disease resistance to BW. It was previously shown that the expression of *AtEFR* in tomato confers increased resistance to *R. solanacearum* (Lacombe et al., 2010). *R. solanacearum* UY031 strain was used for the inoculation assays, and as expected, disease development was lower in partial resistant clone 09309.6 as compared to susceptible INIA Iporá plants. Interestingly transformed AtEFR genotypes had enhanced resistance to BW compared to wild-type plants with INIA Iporá AtEFR and clone 09509.6 AtEFR lines showing an average reduction of 68% and 77%, respectively, in AUDPC. These differences could be related to insertion effects and/or copy number that could lead to gene expression differences. These results, however, clearly showed that *AtEFR* expression conferred resistance to BW in both genotypes. Interestingly, the genotype background is apparently important since differences were observed between them. Clone 09509.6 AtEFR plants were the most resistant, demonstrating that the effect of *AtEFR* expression can be enhanced when coupled with quantitative resistance traits introgressed by classical breeding. It is important to note that all assays were performed under conditions favorable for bacterial multiplication, using high inoculum loads and more severe conditions than natural field infections. Therefore, these results are promising for the development of cultivars with enhanced resistance to BW.

Under cool climatic conditions, plants can harbor bacteria without exhibiting symptoms, resulting in latent infection in vascular tissues of the progeny tubers (Hayward, 1991; Priou et al., 1999). This could lead to outbreaks if infected tubers are used at milder weather conditions or planted in pathogen-free areas. In the absence of resistant varieties, one of the most effective means of control is the use of healthy plant material. In most countries, BW is considered as a quarantine disease; thus, seed certification programs have a zero-tolerance policy for the disease (Priou et al., 2010). In addition, evaluation of asymptomatic latent infections should also be considered in breeding programs to ensure the selection of truly resistant germplasm (Priou et al., 2005).

In this work, we performed the evaluation of resistant AtEFR potato plants for latent *R. solanacearum* infection. Assays revealed the presence of latent bacteria either in stem or tuber samples at low concentrations (10^2^–10^3^ cfu⋅mL^-1^). Yao and Allen (2006) established that virulence factors of the bacterium normally are produced when cell density surpasses 10^9^ cfu⋅g^-1^ of host tissue, while Minh Tran et al. (2016) noted that, in water-transporting xylem vessels, bacteria multiply rapidly, reaching over 10^8^ cfu⋅mL^-1^. This is consistent with the absence of external symptoms in foliage and stems in our experiments. When tubers carrying bacteria (10^2^–10^3^ cfu⋅mL^-1^) were used as seeds, potato plants did not show wilting symptoms. Moreover, no bacteria could be found in either wild-type or transformed plants tubers. Since no differences were observed between genotypes, the absence in symptoms could be related to low bacterial initial titers, which did not lead to wilting symptoms nor infection of tubers. To assess this, controlled inoculated soil and tubers at different concentrations will be evaluated in future experiments to determine the threshold of initial bacterial load necessary to develop BW in both genotypes. This result could also be related to the growing conditions; thus, challenging plants in a more severe bacterial prone environment will also be performed.

Finally, field assays under biosafety restrictions are planned in order to evaluate agronomic traits of both genotypes. It would be interesting to assess the response of this genotypes in endemic *R. solanacearum* areas, as well.

AtEFR plants showed resistance to BW in both genotypes. Moreover, breeding clone 09509.6 AtEFR lines showed an enhanced response, indicating that conventionally derived genotypes with partial resistance can be combined with genetic engineering strategies. While gene-for-gene resistance can be broken down by rapidly evolving pathogens, it should be less likely for pathogens to evolve to evade recognition by PRRs given the conserved and essential nature of PAMPs. Furthermore, the combination of a PAMP receptor with quantitative traits presumably having different mechanism of pathogen suppression may prove more durable resistance than using either approach alone. The employed strategies may constitute important elements toward an integrated control of BW in potato.

## Author Contributions

MD-R, FV, CZ, GG, and MS conceived and designed the experiments. FB, CS, SM, LS, VF, and MS performed the experiments. FB, CS, and VF analyzed the data. FB, CS, VF, MS, GG, FV, CZ, and MD-R discussed the findings and interpreted the results. FB, CS, and MD-R wrote the manuscript. All authors have read and approved the final manuscript.

## Conflict of Interest Statement

The authors declare that the research was conducted in the absence of any commercial or financial relationships that could be construed as a potential conflict of interest. The reviewer ADSP and handling Editor declared their shared affiliation.
