# Supplementary material for: Enhanced Bacterial Wilt Resistance in Potato Through Expression of Arabidopsis EFR and Introgression of Quantitative Resistance from Solanum commersonii
Source: Front Plant Sci. 2017 Sep 25;8:1642. doi: 10.3389/fpls.2017.01642 (PMC5627020; doi:10.3389/fpls.2017.01642)
Supplement: Supplementary file 1 [file Presentation_1.PDF]

Supplementary Material

Enhanced Bacterial Wilt resistance in potato through expression of Arabidopsis EFR and introgression of quantitative resistance from *Solanum commersonii*

Boschi F.[1], Schvartzman C.[2], Murchio S.[2], Ferreira V.[3], Siri M.I.[3], Galván G.A.[4], Smoker M.[5], Zipfel C.[5], Vilaró F.[2], and Dalla-Rizza M.[2*]

[1] Instituto Nacional de Semillas (INASE), Canelones, Uruguay

[2] Laboratorio de Proteínas, Unidad de Biotecnología, Instituto Nacional de Investigación Agropecuaria (INIA), Canelones, Uruguay

[3] Cátedra de Microbiología, Departamento de Biociencias, Facultad de Química, Universidad de la República, Montevideo, Uruguay

[4] Departamento de Producción Vegetal, Centro Regional Sur (CRS), Facultad de Agronomía, Universidad de la República, Canelones, Uruguay

[5]The Sainsbury Laboratory, Norwich Research Park, Norwich, UK

*** Correspondence:**
mdallarizza@inia.org.uy

1 Supplementary Figures and Tables

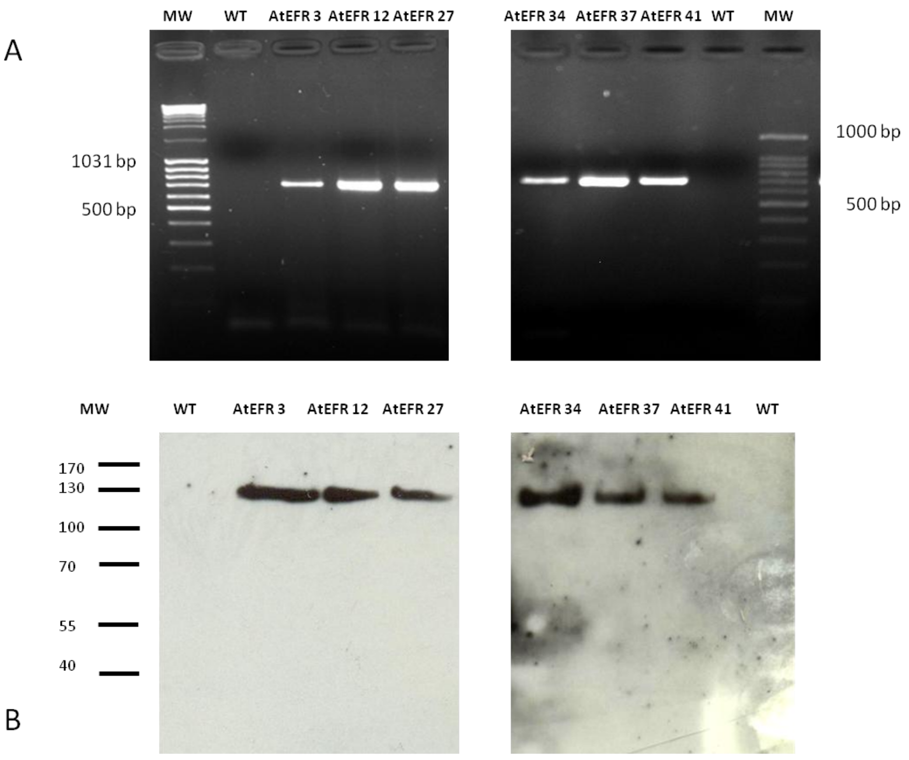

Figure S1. Transgenic expression of AtEFR A. Full PCR gel of *AtEFR* gene detection of transgenic potato lines. left INIA Iporá, right clone 09509.6 **B.** Full Blot of AtEFR protein expression using an anti-HA antibody detecting the fusion protein.

Table S1. Latent *R. solanacearum* infection in stem and tuber of AtEFR potato lines. Colony counts per gram of tissue are expressed as mean ± Standard Error. Basal stems were collected after 28 dpi. Averages of repeats correspond to two independent experiments. First harvested Tubers correspond to those harvested 90 dpi. After plate counting, tubers were used as seeds and tubers were collected after 90 days (second harvested tubers).

	Average plate count CFU.ml^{-1}		
**Event**	**Stem**	**Tuber**	
		**1st harvest**	**2nd harvest**
**INIA Iporá**			
AtEFR 3	$2.4\ x10^{3} \pm 0.1\ x10^{3}$	< 10	< 10
AtEFR 12	$1.1x10^{3} \pm 0.4\ x10^{3}$	$8,8x10^{1} \pm 6,1x10^{1}$	< 10
AtEFR 27	$2.1x10^{3} \pm 0.4\ x10^{3}$	< 10	< 10
WT	$2.2x10^{3} \pm 0.4\ x10^{3}$	$1.9\ x10^{3} \pm 0.1\ x10^{2}$	< 10
**Clone 09509.6**			
AtEFR 34	$4.7x10^{2} \pm 2.6\ x10^{2}$	$3.8x\ 10^{2} \pm 1.7\ x10^{2}$	< 10
AtEFR 37	$4.1x10^{2} \pm 1.8\ x10^{2}$	$2.9x10^{2} \pm 1.5\ x10^{2}$	< 10
AtEFR 41	$1.4x10^{2} \pm 0.7\ x10^{2}$	$3,3\ x10^{2} \pm 2.8x10^{2}$	< 10
Wt	$1.7x10^{3} \pm 0.7\ x10^{2}$	$7.5x10^{2} \pm 0.1\ x10^{2}$	< 10
